# Pyridostigmine Bromide Pills and Pesticides Exposure as Risk Factors for Eye Disease in Gulf War Veterans

**DOI:** 10.3390/jcm12062407

**Published:** 2023-03-21

**Authors:** Lauren E. Truax, Jaxon J. Huang, Katherine Jensen, Elyana V. T. Locatelli, Kimberly Cabrera, Haley O. Peterson, Noah K. Cohen, Simran Mangwani-Mordani, Andrew Jensen, Raquel Goldhardt, Anat Galor

**Affiliations:** 1 Ophthalmology, University of Miami Miller School of Medicine, Miami, FL 33136, USA; 2 Surgical Services, Miami Veterans Affairs Medical Center, Miami, FL 33125, USA; 3 Bascom Palmer Eye Institute, University of Miami, Miami, FL 33136, USA

**Keywords:** pyridostigmine bromide, PB pills, pesticides, Gulf War Illness, dry eye, dry eye symptoms, ocular coherence tomography, macular thickness

## Abstract

To examine associations between the pyridostigmine bromide (PB) pill and/or pesticide exposure during the 1990–1991 Gulf War (GW) and eye findings years after deployment. A cross-sectional study of South Florida veterans who were deployed on active duty during the GW Era (GWE). Information on GW exposures and ocular surface symptoms were collected via standardized questionnaires and an ocular surface examination was performed. Participants underwent spectral domain–ocular coherence tomography (SD-OCT) imaging that included retinal nerve fiber layer (RNFL), ganglion cell layer (GCL), and macular maps. We examined for differences in eye findings between individuals exposed versus not exposed to PB pills or pesticides during service. A total of 40.7% (n = 44) of individuals reported exposure to PB pills and 41.7% (n = 45) to pesticides; additionally, 24 reported exposure to both in the GW arena. Demographics were comparable across groups. Individuals exposed to PB pills reported higher dry eye (DE) symptoms scores (the 5-Item Dry Eye Questionnaire, DEQ-5: 9.3 ± 5.3 vs. 7.3 ± 4.7, *p* = 0.04) and more intense ocular pain (average over the last week: 2.4 ± 2.6 vs. 1.5 ± 1.8, *p* = 0.03; Neuropathic Pain Symptom Inventory modified for the Eye (NPSI-E): 18.2 ± 20.0 vs. 10.8 ± 13.8, *p* = 0.03) compared to their non-exposed counterparts. DE signs were comparable between the groups. Individuals exposed to PB pills also had thicker OCT measurements, with the largest difference in the outer temporal segment of the macula (268.5 ± 22.2 μm vs. 260.6 ± 14.5 μm, *p* = 0.03) compared to non-exposed individuals. These differences remained significant when examined in multivariable models that included demographics and deployment history. Individuals exposed to pesticides had higher neuropathic ocular pain scores (NPSI-E: 17.1 ± 21.1 vs. 11.6 ± 12.9, *p* = 0.049), but this difference did not remain significant in a multivariable model. Individuals exposed to PB pills during the GWE reported more severe ocular surface symptoms and had thicker OCT measures years after deployment compared to their non-exposed counterparts.

## 1. Introduction

Soon after returning home from the Persian Gulf, many Gulf War (GW) veterans (1990–1991) began experiencing a broad constellation of unexplainable symptoms, including fatigue, cognitive dysfunction, muscle aches, diarrhea, and rashes [1,2]. This unsettling phenomenon prompted an effort to identify the cause of the symptoms as well as to formally identify the ailment. Collectively, this complex, chronic disease is now referred to as Gulf War Illness (GWI). One of the models created to define and diagnose GWI is the Kansas criteria. Under the Kansas criteria, six domains were established: fatigue and sleep problems, pain symptoms, neurologic or mood symptoms, gastrointestinal symptoms, respiratory symptoms, and skin symptoms [3]. Today, 34% of GW-deployed veterans meet the Kansas definition of GWI by having at least one chronic symptom in three or more domains, and as many as 60% meet the criteria for diagnosis under the more inclusive Center for Disease Control and Prevention (CDC) guidelines [4,5]. Given the striking proportion of veterans affected, a better understanding of the disease remains a top investigative priority for providers treating this special population.

Chemical exposure has been one postulated factor contributing to GWI as during the GW, service members were exposed to unique toxins not widely encountered in previous conflicts. Specific exposures included pyridostigmine bromide (PB) pills, pesticides, oil well fires, vaccinations (e.g., to anthrax), and nerve gas, among others. While GWI pathophysiology is not fully understood, researchers have hypothesized that some exposures may have acted as neurotoxins that, in combination with genetic predisposition and stress, led to the development of a chronic neuroinflammatory state and the symptoms of GWI [6,7,8]. Of the previously listed exposures, PB pills (given as prophylaxis against nerve gas) and pesticides are most closely studied in connection with GWI [9,10]. In a study of GW veterans who were in Iraq or Kuwait during the conflict, those who met the criteria for GWI (n = 101) were significantly more likely to have received PB pills (OR = 3.50, CI = 1.65 to 7.41, *p* < 0.05) and to have been exposed to pesticides on their skin (OR = 2.07, CI = 1.06 to 4.05, *p* < 0.05) compared to those who did not meet the criteria for GWI (n = 76) [11]. In another study, veterans who took PB pills (n = 200) during the GW had an increased risk of feeling depressed (OR = 1.65, 95% CI = 1.06 to 2.55, *p* = 0.03) and lacking energy (OR = 1.74, 95% CI = 1.14 to 2.64, *p* = 0.01) compared to veterans not exposed to PB pills (n = 92) [12].

Though ocular symptoms are not included as a diagnostic criterion in GWI, our previous studies have found that dry eye (DE) symptoms, including discomfort, dryness, and foreign body sensation, were more severe in deployed GW veterans with GWI symptoms versus controls (deployed without symptoms or non-deployed). Specifically, Ocular Surface Disease Index (OSDI) scores were significantly higher in veterans with GWI symptoms (n = 30) versus controls (n = 41) (41.20 ± 22.92 vs. 27.99 ± 24.03, *p* = 0.01) [13]. In addition to DE symptoms, retinal nerve fiber layer (RNFL) thinning has been noted in deployed GW veterans with GWI symptoms (n = 28) versus controls (n = 38), with the largest difference seen in the inferior RNFL (109.33 ± 26.20 μm vs. 117.00 ± 24.29 μm, *p* = 0.13) [14]. All of these findings point to the potential impact of war time exposures on eye health.

Despite biologic plausibility, there is a paucity of data examining relationships between specific toxins and eye health. To bridge this knowledge gap, we examined relationships between GW exposures reported by veterans to various eye findings, including ocular surface, retinal, and nerve fiber layer metrics.

## 2. Materials and Methods

Study population: We recruited individuals seen in the Miami Veterans Affairs (VA) Hospital eye clinic who were enlisted during the GW (1990–1991). As the goal of the study was to examine the impact of war-time exposures on eye health, we excluded individuals with chronic eye diseases that could confound ocular surface, retinal, and optic nerve testing (anatomical (e.g., pterygium), optic nerve (e.g., glaucoma) or retinal (e.g., diabetic retinopathy) disorders). The study was approved by the Miami Veterans Affairs Institutional Review Board (IRB). All participants signed an informed consent form prior to study participation. The study was conducted in accordance with the principles of the Declaration of Helsinki and complied with the requirements of the United States Health Insurance Portability and Accountability Act. In total, 108 individuals were recruited between 1 October 2020 and 16 May 2022 and underwent an eye examination and filled out an exposure questionnaire.

Exposure data: Each study participant indicated if they were deployed to the GW (1990–1991), if they were deployed to subsequent conflicts in the Middle East (Operation Enduring Freedom, Operation Iraqi Freedom, or another conflict), and what their duties were during deployment. An exposure questionnaire captured self-reported exposures to various agents including PB pills and pesticides/insect repellant. Each exposure question was answered with the options: exposed during the GW 1990–1991, exposed in subsequent deployment, not exposed, or do not know. In this paper, we performed two sets of analyses, first examining individuals who reported ever being exposed to PB pills (irrespective of duration, dose, or time of exposure) as compared to individuals who reported no exposure. We then repeated the analysis comparing individuals who reported any exposure to pesticides while in service compared to those who reported no exposure. Individuals who indicated “do not know” to exposure were grouped with those who reported no exposure.

GWI Symptoms: Participants were categorized as having GWI symptoms in accordance with the Kansas definition. Under this criterion, participants were classified as having GWI symptoms if they had one severe symptom or two moderate symptoms from three of the six domains previously mentioned, irrespective of deployment status [5].

Ocular symptoms: Dry eye (DE) symptoms were assessed using standardized questionnaires: the Ocular Surface Disease Index (OSDI, range 0–100) and the 5-Item Dry Eye Questionnaire (DEQ-5, range 0–22) [15,16]. Ocular pain intensity was graded on a numerical rating scale (NRS) (0 for “no pain”, 10 for “most intense pain imaginable”) and neuropathic pain complaints using the Neuropathic Pain Symptom Inventory modified for the Eye (NPSI-E, total score: range 0–100; sub-score range 0–10) [17]. Convergence insufficiency was assessed using the Convergence Insufficiency Symptoms Survey (CISS, 0–60) [18].

Ocular surface health: A comprehensive ocular surface examination was conducted which included, in the order assessed:

(a) Ocular surface inflammation measured using InflammaDry (Quidel, San Diego). This test measures ocular surface levels of matrix metalloproteinase 9 (MMP9) with the intensity of the pink stripe qualitatively graded as 0 = none, 1 = mild, 2 = moderate, or 3 = severe;
(b) Tear stability assessed by measuring tear break-up time (TBUT) in seconds (three measurements taken in each eye and averaged after instilling 5 μL of fluorescein dye);
(c) Corneal fluorescein staining (cornea divided into five areas and staining graded in each area on a scale of 0 = none to 3 = severe, and summed, according to the National Eye Institute scale) [19];
(d) One drop of proparacaine (Sandoz, Princeton, NJ, USA) instilled into each eye and anesthetized Schirmer’s test performed to quantify tear production at 5 min;
(e) Eyelid and Meibomian gland parameters assessed [19,20]. Specifically, eyelid vascularity was graded on a scale of 0 to 3 (0 = none; 1 = mild engorgement; 2 = moderate engorgement; 3 = severe engorgement) and meibum quality on a scale of 0 to 4 (0 = clear; 1 = cloudy; 2 = granular; 3 = toothpaste; 4 = no meibum extracted). Inferior Meibomian gland (MG) dropout was graded to the Meiboscale based on Lipiscan images (Johnson & Johnson, New Brunswick, NJ, USA).

DE signs were assessed by a provider who was masked to the clinical symptoms for each patient.

Fundus examination and macula and nerve fiber layer imaging: A total of 106 of the 108 study participants who indicated exposure status underwent a dilated fundus examination followed by spectral domain ocular coherence tomography (SD-OCT) imaging that included retinal nerve fiber layer (RNFL), ganglion cell layer (GCL), and macular maps using the Cirrus HD-OCT (Carl Zeiss Meditec Inc., Dublin, CA, USA).

Data analysis: Statistical analysis was performed using the SPSS 28.0 (IBM Corp, Armonk, NY, USA) statistical package. Descriptive statistics were used to summarize patient demographic and clinical information. Chi square test or Fischer’s exact test were used, as appropriate, for categorical variables. Independent samples *T* test was used for continuous variables. The more severe value from each eye was used when examining DE signs. The OCT measurements were analyzed as values from both eyes individually as well as the thinnest and thickest value of either eye. After examining residuals, we conducted multivariable linear regression analyses to examine the robustness of our findings when including potential confounders such as demographics and deployment history. Reported *p*-values were two-tailed and *p* < 0.05 was considered significant.

## 3. Results

### 3.1. Study Population

Our study population consisted of 108 participants who were deployed on active duty during the 1990–1991 GWE and responded to the exposure questionnaire, 69 of whom were deployed to the GW arena. The mean age of the total study population was 55.8 ± 4.8 years, 87% self-identified as male, 58.3% as White, and 34.3% as Hispanic. Overall, 44 participants reported exposure to PB pills, 45 to pesticides, and 24 to both. Table 1 examines demographics, co-morbidities, medications, and GWI symptoms by exposure status. Demographics were matched between exposure groups. A diagnosis of sleep apnea was more common in those exposed to PB pills (75.0% vs. 52.4%, *p* = 0.02) and pesticides (73.3% vs. 53.2%, *p* = 0.04) compared to non-exposed individuals. Individuals who reported exposure to pesticides were more likely to be using antidepressants (38.6% vs. 19.4%, *p* = 0.03) and antihistamines (38.6% vs. 21.0%, *p* = 0.047) compared to non-exposed individuals.

### 3.2. Dry Eye Symptoms and Signs

DE status was assessed in all participants using both symptoms and signs. Overall, individuals exposed to PB pills had significantly greater DE symptoms measured both via the DEQ-5 (9.3 ± 5.3 vs. 7.3 ± 4.7, *p* = 0.04) and OSDI (39.4 ± 26.0 vs. 28.7 ± 21.7, *p* = 0.03). PB pills were also significantly associated with an increased intensity in ocular pain as reported by the NRS (averaged over the last week: 2.4 ± 2.6 vs. 1.5 ± 1.8, *p* = 0.03). Both exposure to PB pills (18.2 ± 20.0 vs. 10.8 ± 13.8, *p* = 0.03) and pesticides (17.1 ± 21.1 vs. 11.6 ± 12.9, *p* = 0.049) were associated with a higher intensity of neuropathic such as eye symptoms, based on the NPSI-E. However, DE signs were similar regardless of exposure status (Table 2).

### 3.3. Fundus Examination and Ocular Coherence Tomography Measures, Grouped by Exposure Status

No overt retinal abnormalities were noted on dilated fundus examination. However, some differences were noted between groups with respect to SD-OCT measures (RNFL, GCL, and overall macular thickness were examined at multiple locations, Table S1 (Supplementary Materials)). There were no significant differences in measurements between groups with respect to NFL or GCL metrics. However, some differences were noted with respect to overall macular thickness, with the most pronounced difference being in the left outer temporal area. Individuals exposed to PB pills had thicker outer temporal measurements (268.5 ± 22.2 μm vs. 260.6 ± 14.5 μm, *p* = 0.03) compared to those unexposed, though these measurements were still within a physiologic range.

SD-OCT measurements with a P value less than 0.10 were also analyzed as the thinnest or thickest value of either eye. The thicker outer temporal macular measurement on OCT again remained the only significant difference between those exposed to PB pills and controls (271.9 *±* 21.5 μm vs. 264.0 *±* 14.7 μm, *p* = 0.03). While no other values were significantly different, PB pill exposure correlated with a general trend of thicker measurement on SD-OCT compared to controls, although all values again remained within a physiologic range.

### 3.4. GWI Symptoms, Grouped by Exposure Status

GWI symptoms were more frequent in individuals who reported a history of PB use (47.7% vs. 29.7%, *p* = 0.06), and less frequent in those who reported pesticides exposure (30.2% vs. 46.7%, *p* = 0.08) compared to non-exposed individuals; however, these numbers did not reach statistical significance.

### 3.5. Multivariable Modeling

Multivariable modeling was performed to examine relationships between exposures and eye findings, while controlling for potential confounders. Independent variables included in forward linear regression models were demographics (age, race, ethnicity, and gender), sleep apnea, PB pills exposure, pesticides exposure, and deployment status. Separate models were run for each of the dependent variables: DEQ-5, OSDI, NRS average score over the last week, NPSI-E, and left outer temporal macular measurement on OCT. In all five models, only PB pills exposure remained statistically significant (Table 3).

Forward stepwise binary logistic regression with demographics (age, race, ethnicity, and gender), sleep apnea, PB pills exposure, pesticides exposure, and deployment status as the independent variables was also modeled with presence of GWI symptoms as the dependent variable. Both deployment and race remained significant in the model (Table 4).

## 4. Discussion

To summarize, we found that veterans who reported taking PB pills during the GWE had more severe DE symptoms (as reported by both the DEQ-5 and OSDI) and ocular pain (as measured by both the NRS and NPSI-E) than their non-exposed counterparts. These findings suggest a correlation between PB pill exposure and ocular symptoms. Interestingly, DE signs were not significantly different between the groups, which is not surprising given the known disconnect between DE symptoms and signs [21]. On SD-OCT, individuals exposed versus non-exposed to PB overall had a higher mean thicknesses on RNFL, macula, and GCL measures, with the difference becoming significant at the left outer temporal macular region and remaining significant when analyzing the thickest value of either eye. These noted differences remained significant in multivariable models that accounted for deployment (yes/no), demographics, and sleep apnea in addition to exposure to PB pills and pesticides. In contrast, correlations between pesticides exposure and eye findings were weaker, with a higher intensity of neuropathic like ocular pain symptoms (via the NPSI-E) on univariable analysis. However, this difference did not remain significant in a multivariable model.

**Figure 1:**
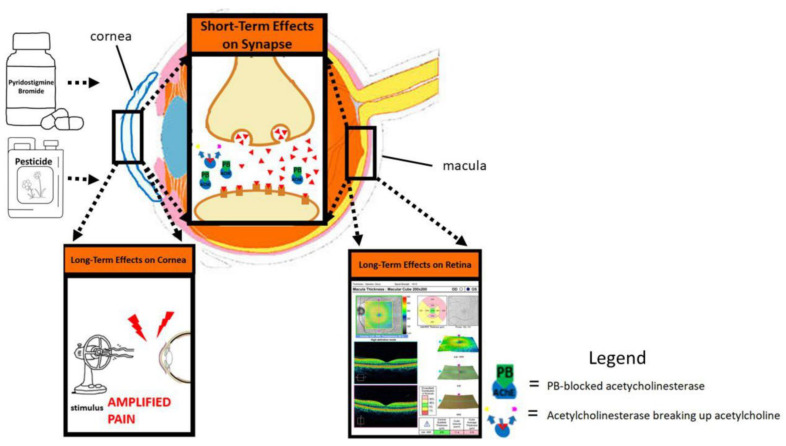
Visual summary of the possible effects of PB pills and pesticides on the cornea and retina.

Both PB pills and most pesticides used during the GW (organophosphates, carbamates, and pyrethroids) are acetylcholinesterase (AChE) inhibitors. PB reversibly binds AChE and inhibits the breakdown of acetylcholine in the synaptic junction, leading to an excess of acetylcholine (ACh) in the central nervous system (CNS), autonomic ganglia of the parasympathetic nervous system (PNS), and neuromuscular junctions [22]. Excess ACh disrupts these systems by hyperstimulation of the muscarinic and nicotinic receptors, and all AchE inhibitors can cause cholinergic toxicity [23]. Symptoms of cholinergic toxicity include salivation, lacrimation, miosis, diaphoresis, abdominal pain, vomiting, diarrhea, and bronchospasm [24]. PB pills were used by military personnel serving in the GW as a prophylactic agent against nerve gas. Nerve gas irreversibly binds AChE, whereas PB reversibly binds AChE, rendering it temporarily unavailable to be bound by nerve gas (Figure 1) [25]. PB was assumed safe as a prophylactic treatment on the basis of its use in treating myasthenia gravis (MG), an autoimmune disease that attacks nicotinic acetylcholine receptors [12]. PB was approved for use by the Federal Drug Administration (FDA) for treatment of MG in 1955 as a means of increasing the amount of available acetylcholine in the synapse and promoting neuromuscular function [25,26]. It was supplied to GW servicemembers as a 21-tablet blister pack and prescribed as one 30-mg tablet three times per day [22]. Per the RAND survey, around 50% (250,000) of the GW in-theater personnel used PB pills [27]. Of those 50% who reported using PB, 95% reported taking three or fewer pills per day for less than one month. The average self-reported use was 20 pills per 30-day period [27]. Those troops who did take PB pills as prescribed reported symptoms of cholinergic toxicity as previously described, which most commonly included nausea, vomiting, diarrhea, and abdominal pain [25]. Additionally, it is estimated that about 66% of on-the-ground, in-theater GW veterans were exposed to or personally used pesticides on their skin, their uniforms, as area sprays/foggers, in pest strips, in fly baits, and to delouse themselves [22,28].

Ocular pain in the setting of PB exposure has not been reported in the MG literature. However, blurred vision and increased lacrimation have been reported side effects of the medication [29]. One potential difference between the PB use in GW veterans versus MG patients is the coupling effect of stress. Previous studies have demonstrated that the interaction between PB and stress impacts on glutamate neurochemistry, microglia activation, and neurogenesis to a degree not seen with PB exposure alone [30,31,32]. It is possible that the PB use coupled with the stressful environment in the GW theater led to alterations in brain regions associated with pain processing, with one manifestation being ocular pain.

Our findings of increased DE symptoms and ocular pain, but similar DE signs, in individuals exposed versus non-exposed to PB pills suggest that ocular symptoms in our cohort were driven predominantly by nerve, and not tear, abnormalities. In fact, it has been increasingly recognized that neuropathic mechanisms underlie ocular pain in some individuals [33]. PB pills may sensitize nerves and contribute to the onset of neuropathic pain elsewhere in the body, as demonstrated in animal models. In one study, cultured rat cerebellar granule cells were exposed to varying concentrations of PB for 24 h. Experimenters found that at concentrations of PB at 500 µM (*p* < 0.05) and 1000 µM (*p* < 0.01), there was a significant efflux of LDH from cells indicating cytotoxicity. TUNEL staining, a technique used to detect morphological changes in apoptosis, demonstrated a dose-dependent apoptotic cell death, with up to 40% of cells containing fragmented DNA at 250 µM. Apoptosis was also indicated by DNA fragmentation on agarose gel electrophoresis at PB concentrations of 50 µM, 100 µM, and 250 µM. Two indicators of apoptosis, cytochrome c release from mitochondria and the activation of caspase-3, were both assessed. For cytochrome c, concentrations of PB as low as 50 µM caused prominent staining on Western blot. Activation of caspase-3 was significantly increased compared to control at PB concentrations of 100 µM (*p* < 0.01) and 250 µM (*p* < 0.001). A dose-dependent generation of intracellular reactive oxygen species (ROS) was observed, with a significant increase in ROS generation detected even at a PB concentration as low as 30 µM (*p* < 0.01). These results indicate that apoptosis in cerebellar granule cells can be caused by low concentrations of PB [34]. The exposure to AchE inhibitors may similarly affect some humans, inducing neuronal degeneration of the cerebellum and other regions of the brain associated with pain-processing. Our current findings, namely, that veterans exposed to PB pills, and less strongly to pesticides, while in the GW theater had higher levels of ocular pain compared to those not exposed strengthens the hypothesis that past PB exposure adversely affects how ocular surface stimuli are processed, with a propensity towards ocular pain.

In addition, we also found that individuals exposed to PB pills had thicker SD-OCT measures than their non-exposed counterparts, although clinically, no overt abnormalities were noted on examination. Several possibilities may underlie the noted findings including the presence of neuroinflammation or reactive gliosis. In fact, neuroinflammation has been reported to occur after PB exposure in a number of studies [7,35,36]. One study exposed rats to PB, DEET, permethrin, and stress in the form of restraint for 15 min a day for 28 days and found higher levels of HMGB1 in the brain (an activator of the complement system) in exposed rats compared to control rats not exposed to chemicals or stress [35]. In addition, stressed rats exposed to chemicals had a greater number of hypertrophied microglia (another measurement of neuroinflammation) in the brain compared to control rats [35]. Neuroinflammation has also been found to be a component of GWI in humans, albeit not specifically connected to PB exposure. One study investigated neuroinflammation in GWI using a radioligand to detect a neuroinflammatory marker, 18-kDA translocator protein (TPSO) on proton emission tomography. TPSO is a mitochondrial protein that is scarce in healthy nervous system tissue, but is upregulated by activated microglia and proinflammatory cytokines in neuroinflammatory processes [37]. Veterans with GWI (n = 15) showed a statistically significant (*p* < 0.05) elevation of radioligand detecting TPSO in the precuneus, prefrontal, and primary and somatosensory cortices compared to veterans without GWI (n = 8), though deployment status was not considered in selecting participants for this study [7]. It is possible that similar processes also occur in the retina and can be detected by SD-OCT. It is important to note, however, that in a prior study, we found that RNFL measures were thinner in deployed GW veterans with GWI symptoms versus controls (deployed without symptoms or not deployed), with a mean 6.55% decrease in inferior RNFL thickness [14]. We hypothesize that exposures in the GW arena may have both neuroinflammatory and neurodegenerative consequences, which could present as thickening or thinning in RNFL and/or macular metrics and that the contribution of these two processes may change SD-OCT measures over time. Longitudinal studies are needed to test this hypothesis.

Both PB pills and pesticides exposures have been linked to other aspects of GWI in prior studies. A study of 604 GW veterans examined relationships between neurotoxin exposure and four GWI domains (neurocognitive/mood, gastrointestinal, fatigue/sleep, and pain) [10]. Exposure to both pesticides (OR = 4.13, 95% CI = 1.78–9.57) and PB pills (OR = 2.28, 95% CI = 1.02–5.09) were strongly associated with pain severity. Pesticides were also associated with difficulties in concentrating (OR = 2.29, 95% CI = 1.04–5.02) and recall (OR = 2.18, 95% CI = 1.04–4.56), difficulty with sleep (OR = 3.06, 95% CI = 1.19–7.89), unrefreshing sleep (OR = 3.13, 95% CI = 1.09–8.98), and joint pain (OR = 2.46, 95% CI = 1.04–5.82). PB pill use was associated with depression (OR = 2.68, 95% CI = 1.28–5.60) [10]. In our study, we did not examine sleep quality but did find that individuals exposed to PB and pesticides were significantly more likely to be diagnosed with sleep apnea compared to their respective controls. This association has also been described in the setting of pesticide use outside military exposure. One prospective study examined 1569 pesticide applicators in the US farming community who reported exposure to any one or more of 63 pesticides [38]. Of those pesticides, an association was noted between sleep apnea and carbofuran, a carbamate pesticide (OR = 1.83, *p* = 0.0002) [38]. This suggests that exposures during the GW arena may impact the frequency of co-morbidities beyond the eye, including sleep and mental health.

As with all studies, our findings must be considered bearing in mind the limitations, including a population restricted to the south Florida region and a reliance on recall to collect exposure data. Participants may have varying recollection of events during their service in the GW, as well as uncertainty surrounding the specific chemicals that were used around the camps or as treatment on uniforms. Thus, recall bias may be present in our study. Additionally, some individuals in this study indicated that they were exposed to both PB and pesticides. The grouping of pesticides into one category limits our ability to identify whether one particular type, or a combination of pesticides, has a more significant effect on ocular symptoms and signs. Furthermore, information on the duration of exposure is lacking, and this may impact on our results. Future studies will need to build on our findings, with a more detailed capture of types and duration of pesticides exposure to bridge the current knowledge gaps. In addition, other uncollected confounders (diet, exposure to stress) may have influenced our data. Despite these limitations, this study is an important step in examining the link between GW and eye health. A better understanding of this connection can help providers screen, diagnose, and treat patients which can translate to improvements in quality of life. Longitudinal studies with increased sample sizes in diverse populations are necessary to determine if our findings are reproducible across centers, and investigate whether other common exposures impact on eye health, such as nerve gas and oil well fires. Furthermore, more data are needed to determine the utility of OCT findings as biomarkers for GW exposures/illness.

## Figures and Tables

**Table 1:** Demographics, co-morbidities, and medication use in the study population, divided by exposure status.

Mean ± SD or Percent Frequency	PB Pills Exposed (n = 44)	PB Pills Control (n = 64)	PB Pills *p*-Value	Pesticides Exposed (n = 45)	Pesticides Control (n = 63)	Pesticides *p*-Value
Demographics						
Age	55.5 ± 4.9	56.0 ± 4.7	0.65	55.4 ± 4.6	56.0 ± 4.9	0.52
Male Gender	90.9%	84.4%	0.32	88.9%	85.7%	0.63
White	59.1%	57.8%	0.77	57.8%	57.1%	0.95
Hispanic	36.4%	32.8%	0.70	37.8%	31.7%	0.52
Comorbidities						
Diabetes	13.6%	21.9%	0.28	20.0%	17.5%	0.74
Hypertension	34.1%	41.3%	0.45	42.2%	35.5%	0.48
Hyperlipidemia	39.5%	50.0%	0.29	43.2%	47.6%	0.65
Sleep Apnea	75.0%	52.4%	0.02 *	73.3%	53.2%	0.04 *
Depression	50.0%	38.1%	0.22	44.4%	41.9%	0.80
PTSD	41.9%	30.2%	0.22	43.2%	29.0%	0.13
Arthritis	42.9%	27.4%	0.10	37.2%	31.1%	0.52
BPH	9.3%	17.5%	0.24	15.9%	12.9%	0.66
Medication use						
Fish oil	29.5%	21.0%	0.31	34.1%	17.7%	0.05
Multivitamin	38.6%	34.9%	0.69	40.9%	33.3%	0.42
NSAID	38.6%	38.7%	0.99	45.5%	33.9%	0.23
Gabapentin	20.5%	9.7%	0.12	15.9%	12.9%	0.66
Aspirin	20.5%	21.0%	0.95	29.5%	14.5%	0.06
Statin	47.7%	48.4%	0.95	52.3%	45.2%	0.47
Betablocker	13.6%	17.7%	0.57	13.6%	17.7%	0.57
Antidepressant	34.1%	22.6%	0.19	38.6%	19.4%	0.03 *
Antianxiety	20.5%	16.1%	0.57	22.7%	14.5%	0.28
Antihistamine	31.8%	25.8%	0.50	38.6%	21.0%	0.047 *
GWI symptoms	47.7%	29.7%	0.06	30.2%	46.7%	0.08
Deployment	91.0%	45.3%	<0.001 *	75.6%	55.6%	0.03 *

GWI: Gulf War Illness; PB: pyridostigmine bromide. * Statistically significant difference at *p* value < 0.05.

**Table 2:** Dry eye symptoms and signs in individual groups by exposure status.

Mean ± SD	PB Pills Exposed (n = 44)	PB Pills Control (n = 64)	PB Pills *p*-Value	Pesticides Exposed (n = 45)	Pesticides Control (n = 63)	Pesticides *p*-Value
Symptoms						
DEQ-5, range 0–22	9.3 ± 5.3	7.3 ± 4.6	0.04 *	8.2 ± 5.2	8.1 ± 4.8	0.90
OSDI, range 0–100	39.4 ± 26.0	28.7 ± 21.7	0.03 *	35.2 ± 25.6	31.6 ± 23.0	0.50
NRS right now, range 0–10	2.2 ± 2.8	1.0 ± 1.6	0.01 *	1.5 ± 2.3	1.5 ± 2.2	0.96
NRS average over past week, range 0–10	2.4 ± 2.6	1.5 ± 1.8	0.03 *	1.6 ± 2.1	2.0 ± 2.3	0.45
NRS worse over past week, range 0–10	3.1 ± 3.3	1.6 ± 2.0	0.01 *	2.1 ± 2.9	2.2 ± 2.6	0.79
NPSI-E total, range 0–100	18.2 ± 20.0	10.8 ± 13.8	0.03 *	17.1 ± 21.1	11.6 ± 12.9	0.049 *
CISS	23.2 ± 14.7	18.4 ± 12.3	0.08	20.6 ± 14.6	20.1 ± 12.7	0.84
Signs						
MMP9, range 0–3	1.3 ± 1.6	1.1 ± 1.0	0.65	1.3 ± 1.7	1.1 ± 0.9	0.53
TBUT, seconds	8.4 ± 4.0	9.2 ± 4.6	0.32	8.4 ± 4.8	9.2 ± 4.1	0.39
Corneal staining, range 0–15	1.6 ± 2.3	1.0 ± 1.6	0.08	1.4 ± 2.6	1.1 ± 1.4	0.40
Schirmer test, mm wetting at 5 min	11.8 ± 7.1	14.8 ± 9.6	0.09	14.3 ± 8.5	13.0 ± 9.0	0.45
Eyelid vascularity, range 0–3	0.6 ± 0.8	0.5 ± 0.7	0.56	0.7 ± 0.8	0.5 ± 0.7	0.32
Meibum quality, range 0–4	1.5 ± 1.2	1.2 ± 1.0	0.17	1.5 ± 1.1	1.2 ± 1.0	0.15
Meibomian gland dropout, range 0–4	2.0 ± 1.4	1.9 ± 1.2	0.75	2.1 ± 1.1	1.8 ± 1.4	0.24

SD: standard deviation; PB: pyridostigmine bromide; DEQ-5: Dry Eye Questionnaire 5; OSDI: Ocular Surface Disease Index; NRS: Numerical Rating Scale; NPSI-E: Neuropathic Pain Symptom Inventory modified for the Eye; CISS: Convergence Insufficiency Symptoms Survey; MMP9: matrix metalloproteinase 9; TBUT: tear break-up time. * Statistically significant difference at *p* value < 0.05.

**Table 3:** Linear regression for predictors of significant ocular markers.

Predictor	Standardized Coefficients β	*p*-Value
DEQ-5		
PB pills	0.201	0.04 *
Pesticides **	--	0.78
OSDI		
PB pills	0.218	0.03 *
Pesticides **	--	0.85
NRS average pain intensity over one week recall		
PB pills	0.228	0.02 *
Pesticides **	--	0.21
NPSI-E		
PB pills	0.219	0.03 *
Pesticides **	--	0.22
Macular outer temporal OCT measurement, left eye		
PB pills	0.207	0.03 *
Pesticides **	--	0.15

PB: pyridostigmine bromide; DEQ-5: Dry Eye Questionnaire 5; OSDI: Ocular Surface Disease Index; NRS: Numerical Rating Scale; NPSI-E: Neuropathic Pain Symptom Inventory modified for the Eye. * Statistically significant difference at *p* value < 0.05. ** Variables were not predictors in the final model for significant ocular markers and thus only have *p*-values calculated. They are included in this table for completeness.

**Table 4:** Logistic regression for predictors of GWI symptoms.

Predictor	β	SE	Wald Statistic	*p*-Value	OR	OR 95% CI
Deployment	1.02	0.46	4.96	0.03 *	2.79	1.13–6.86
Race	0.87	0.43	4.08	0.04 *	2.40	1.02–5.60
PB pills **	--	--	--	0.42	--	--
Pesticides **	--	--	--	0.21	--	--

SE: standard error; OR: odds ratio; CI: confidence interval; PB: pyridostigmine bromide. * Statistically significant difference at *p* value < 0.05. ** Variables were not predictors in the final model for significant ocular markers and thus only have *p*-values calculated. They are included in this table for completeness.

## Data Availability

The data presented in this study are available upon reasonable request to the corresponding author.

## Supplementary Materials

The following supporting information can be downloaded at: https://www.mdpi.com/article/10.3390/jcm12062407/s1, Table S1: OCT measurements grouped by exposure status.
