# Supplementary material for: Pyridostigmine Bromide Pills and Pesticides Exposure as Risk Factors for Eye Disease in Gulf War Veterans
Source: J Clin Med. 2023 Mar 21;12(6):2407. doi: 10.3390/jcm12062407 (PMC10059791; doi:10.3390/jcm12062407)
Supplement: Supplementary file 1 [file jcm-12-02407-s001.zip › jcm-2229837-supplementary.pdf]

Supplementary Table S1. OCT measurements grouped by exposure status.

Mean±SD	PB Pills Exposed (n=44)	PB Pills Control (n=62)	*P*-value	Pesticides Exposed (n=44)	Pesticides Control (n=62)	*P*-value
**RNFL, µm**						
**Right eye**						
Signal strength	0.9±0.2	0.9±0.1	0.78	0.9±0.1	0.9±0.1	0.33
Superior	115.8±18.9	112.4±15.4	0.32	115.0±17.4	113.0±16.7	0.55
Nasal	75.4±12.9	71.8±11.4	0.14	71.9±11.6	74.3±12.4	0.31
Inferior	124.8±20.4	119.5±20.4	0.20	122.8±18.4	120.9±22.0	0.63
Temporal	64.8±13.6	63.4±12.3	0.58	63.6±11.1	64.3±14.0	0.77
Average	95.3±12.6	91.8±10.4	0.12	93.4±10.0	93.2±12.5	0.92
Average C/D	0.5±0.2	0.5±0.2	0.85	0.5±0.1	0.5±0.2	0.44
**Left eye**						
Signal strength	0.9±0.1	0.9±0.1	0.98	0.9±0.1	0.9±0.1	0.52
Superior	122.0±18.3	115.7±22.5	0.13	119.3±16.6	117.6±23.7	0.68
Nasal	73.0±13.3	70.4±11.8	0.29	69.7±11.8	72.7±12/8	0.22
Inferior	125.1±21.2	118.0±18.8	0.07†	122.5±21.2	119.9±19.3	0.53
Temporal	65.2±21.5	59.6±11.7	0.09†	65.3±21.2	59.5±12.1	0.08
Average	95.2±12.1	91.4±11.0	0.10	93.1±10.5	92.9±12.4	0.94
Average C/D	0.5±0.2	0.5±0.2	0.86	0.5±0.2	0.5±0.2	0.48
**Macula, µm**						
**Right eye**						
Signal strength	0.9±0.1	0.9±0.1	0.75	0.9±0.1	0.9±0.1	0.88
Central	264.1±19.3	258.1±28.1	0.23	257.9±25.0	262.5±24.8	0.35
Inner superior	325.6±16.1	320.5±17.2	0.13	321.0±15.4	323.8±17.8	0.40
Outer superior	280.1±15.2	276.1±15.1	0.19	275.2±11.8	279.6±17.0	0.14
Inner nasal	329.0±17.3	322.2±18.5	0.06†	325.0±18.3	325.1±18.3	0.98
Outer nasal	297.2±20.4	293.0±20.2	0.31	291.9±17.2	296.8±17.2	0.22
Inner inferior	322.4±15.5	316.3±17.1	0.06†	318.0±15.3	319.5±17.6	0.64
Outer inferior	269.1±15.0	263.9±15.1	0.08†	264.6±13.1	267.0±16.5	0.42
Inner temporal	313.7±14.5	309.5±16.9	0.18	311.3±15.9	311.2±16.3	0.96
Outer temporal	262.9±19.4	262.2±13.9	0.84	261.8±12.0	263.0±18.9	0.70
**Left eye**						
Signal strength	0.9±0.1	0.9±0.1	0.83	0.9±0.1	0.9±0.1	0.59
Central	265.7±30.4	258.7±32.8	0.27	263.9±33.2	260.0±31.0	0.53
Inner superior	325.2±17.0	321.6±19.3	0.33	322.8±14.8	323.2±20.7	0.91
Outer superior	279.8±15.4	276.0±15.4	0.21	276.4±12.0	278.4±17.5	0.51
Inner nasal	326.1±17.6	324.0±20.4	0.57	325.8±16.7	324.2±20.9	0.69
Outer nasal	295.3±19.4	293.3±18.1	0.59	293.8±13.6	294.3±21.5	0.87
Inner inferior	319.8±16.5	316.6±19.7	0.38	317.6±16.4	318.2±19.8	0.88
Outer inferior	270.4±17.4	264.9±15.6	0.09†	267.2±16.2	267.2±16.9	0.996
Inner temporal	313.1±15.2	308.7±18.7	0.21	310.2±15.6	310.8±18.6	0.87
Outer temporal	268.5±22.2	260.6±14.5	0.03*†	262.1±17.8	265.2±18.8	0.39
**GCL, µm**						
**Right eye**						
Superior	82.6±7.2	79.3±12.0	0.12	81.4±7.1	80.1±12.3	0.53
Superior nasal	83.5±8.6	79.0±14.6	0.07	82.3±7.9	79.8±15.1	0.34
Inferior nasal	81.9±8.7	78.7±10.5	0.10	81.4±7.6	79.1±11.2	0.24
Inferior	79.9±8.9	77.0±10.9	0.14	79.9±6.8	77.0±11.9	0.16
Inferior temporal	82.7±7.0	79.7±10.0	0.09	82.2±7.0	80.0±10.1	0.24
Superior temporal	81.1±7.0	78.2±10.0	0.11	80.0±6.7	79.0±10.3	0.55

Average GCL+IPL thickness	82.0±7.3	78.8±10.5	0.09	81.2±6.6	79.4±11.0	0.34
**Left eye**						
Superior	81.6±11.2	81.0±8.9	0.78	81.0±10.6	81.5±9.5	0.83
Superior nasal	80.5±16.4	81.6±10.4	0.67	82.2±9.0	80.3±15.6	0.47
Inferior nasal	80.0±12.9	79.7±10.0	0.92	80.5±9.1	79.3±12.7	0.59
Inferior	79.0±10.0	77.5±10.0	0.45	78.7±7.2	77.8±11.6	0.65
Inferior temporal	81.4±7.7	79.6±9.2	0.28	80.8±7.0	80.1±9.7	0.69
Superior temporal	81.2±7.5	79.3±8.2	0.22	80.2±6.9	80.0±8.6	0.91
Average GCL+IPL thickness	80.8±9.2	79.9±8.9	0.57	80.6±7.3	80.0±10.1	0.77

RNFL: retinal nerve fiber layer, PB: pyridostigmine bromide; C/D: cup-to-disc ratio; GCL: ganglion cell layer, IPL: inner plexiform layer

*Statistically significant difference at P value <0.05

†P value<0.01 and further analyzed as thinnest and thickest value of either eye
